# Comparative metabolomics of muscle interstitium fluid in human trapezius myalgia: an in vivo microdialysis study

**DOI:** 10.1007/s00421-013-2716-6

**Published:** 2013-09-28

**Authors:** J. Hadrévi, B. Ghafouri, A. Sjörs, H. Antti, B. Larsson, A. G. Crenshaw, B. Gerdle, F. Hellström

**Affiliations:** 1 Section for Anatomy, Department of Integrative Medical Biology, Umeå University, 901 87 Umeå, Sweden; 2 Department of Occupational and Public Health Sciences, Faculty of Health and Occupational Studies, Centre for Musculoskeletal Research, University of Gävle, 907 12 Umeå, Sweden; 3 Rehabilitation Medicine, Department of Medicine and Health Sciences (IMH), Faculty of Health Sciences, Pain and Rehabilitation Centre, Linköping University, County Council of Östergötland, 581 85 Linköping, Sweden; 4 Occupational and Environmental Medicine, Department of Clinical and Experimental Medicine, Faculty of Health Sciences, Centre of Occupational and Environmental Medicine, Linköping University, County Council of Östergötland, 581 85 Linköping, Sweden; 5 Institute of Stress Medicine, Carl Skottsbergs Gata 22B, 413 19 Gothenburg, Sweden; 6 Department of Chemistry, Faculty of Science and Technology, Umeå University, 901 85 Umeå, Sweden

**Keywords:** Metabolomics, Trapezius myalgia, Microdialysis, Repetitive work, Recovery, GC–MS, Metabolites

## Abstract

**Purpose:**

The mechanisms behind trapezius myalgia are unclear. Many hypotheses have been presented suggesting an altered metabolism in the muscle. Here, muscle microdialysate from healthy and myalgic muscle is analysed using metabolomics. Metabolomics analyse a vast number of metabolites, enabling a comprehensive explorative screening of the cellular processes in the muscle.

**Methods:**

Microdialysate samples were obtained from the shoulder muscle of healthy and myalgic subjects that performed a work and stress test. Samples from the baseline period and from the recovery period were analysed using gas chromatography—mass spectrometry (GC–MS) together with multivariate analysis to detect differences in extracellular content of metabolites between groups. Systematic differences in metabolites between groups were identified using multivariate analysis and orthogonal partial least square discriminate analysis (OPLS-DA). A complementary Mann–Whitney *U* test of group difference in individual metabolites was also performed.

**Results:**

A large number of metabolites were detected and identified in this screening study. At baseline, no systematic differences between groups were observed according to the OPLS-DA. However, two metabolites, l-leucine and pyroglutamic acid, were significantly more abundant in the myalgic muscle compared to the healthy muscle. In the recovery period, systematic difference in metabolites between the groups was observed according to the OPLS-DA. The groups differed in amino acids, fatty acids and carbohydrates. Myristic acid and putrescine were significantly more abundant and beta-d-glucopyranose was significantly less abundant in the myalgic muscle.

**Conclusion:**

This study provides important information regarding the metabolite content, thereby presenting new clues regarding the pathophysiology of the myalgic muscle.

## Introduction

Little is known about the pathophysiological mechanisms behind work-related disorders, especially regarding chronic trapezius myalgia. In an attempt to shed light on the biochemistry of myalgic muscle, a number of studies have employed methodologies such as muscle biopsies and microdialysis. Results obtained with these methods have not been conclusive in explaining the peripheral mechanisms of pain, but a generalisation to be made is that evidence favours metabolic and algesic disturbances in the muscle tissue (Gerdle and Larsson 2012; Visser and van Dieen 2006).

Biopsy samples of myalgic muscle have shown morphological irregularities. Slow contracting type 1 fibres were shown to occur to a larger extent in myalgic compared to healthy muscle (Kadi et al. 1998b; Lindman et al. 1991; Andersen et al. 2008c). Also present in myalgic muscle were aggregations of mitochondria within muscle cells such as increased prevalence of moth eaten and ragged red fibres (Larsson et al. 1988; Kadi et al. 1998b). These aggregations are indicative of mitochondrial disturbances and believed to be evidence of altered metabolism in the myalgic muscle. A recent finding of reduced metabolic protein production for myalgic versus healthy muscle was discussed in the context of differences in mitochondrial function between groups (Sjøgaard et al. 2013). The mitochondrial alterations may via different mechanisms be linked to pain; for example, increased content of intramuscular fat can result in release of pro-inflammatory substances (cytokines and leptin), increased concentration of lactate with activation of acid-sensing ion channel 3 (ASIC-3) and reactive oxygen species (ROS) generation which directly or indirectly interact with the nociceptive system (for references see Gerdle et al. 2013a, b).

Thus, a link between mitochondrial function and perceived pain has been speculated. However, some results suggest that moth eaten fibres and ragged red fibres are related to work exposure, per se, and not to the pain (Kadi et al. 1998a; Larsson et al. 2000b). These types of morphological disturbances have also been related to muscle turnover due to their high prevalence in muscle dystrophies.

Changes in metabolism have been addressed using the microdialysis (MD) technique. MD allows for in vivo collection of extracellular fluid from the interstitium of muscles, thereby providing access to the chemical changes in the muscle (Ungerstedt 1991). Comparisons between myalgic and healthy trapezius muscle at rest, during low-load repetitive work and recovery have been studied (Flodgren et al. 2005, 2010; Rosendal et al. 2004a, b, 2005a, b; Sjogaard et al. 2010). In most of these studies, patients with chronic trapezius myalgia were shown to have increased interstitial levels of lactate and pyruvate compared to healthy subjects. Even though increased anaerobic metabolism has been suggested as an explanation to these findings, the results cannot be explained by insufficiencies in muscle oxygenation (Sjogaard et al. 2010; Larsson et al. 2008; Andersen et al. 2010). Furthermore, expressions of lactate and pyruvate alone may not describe the entire picture in the myalgic muscle.

In our opinion, a more comprehensive assessment of the metabolism in the muscle environment could give credence to discrepancies seen in the above-mentioned studies, as well as provide new clues about the mechanism behind chronic trapezius myalgia. Metabolomics using gas chromatography mass spectrometry (GC–MS) is a technique that screens for a large number of metabolites simultaneously. This approach is based on the systematic study of the unique small-molecule chemical fingerprints that specific cellular processes leave behind. The metabolites are the end products of the cellular processes. With metabolomics, a snap-shot of the physiology of the cell is provided. This technology, ideal for identifying diagnostic biomarkers, consists of two sequential steps: (1) an experimental technique, based on MS or nuclear magnetic resonance spectroscopy, designed to profile low molecular weight compounds, and (2) multivariate data analysis (Dunn and Ellis 2005). Metabolomic analysis of biofluids (e.g. microdialysates) or tissues (e.g. biopsies) has been successfully used in the fields of physiology, diagnostics, functional genomics, pharmacology, toxicology and nutrition (Dunn and Ellis 2005). Metabolomics has not previously been applied when investigating chronic myalgia, but could add a new dimension in understanding the underlying mechanisms.

The aim of the present study was to use metabolomics to determine metabolites in the trapezius muscle for a group of patients with trapezius myalgia in comparison to a healthy control group. The MD technique was used to collect dialysate samples before and after a standardised work and stress test. This allowed for assessment of group differences as well as differences in metabolite content following a work and stress task.

## Methods

### Subjects

**Fig. 1:**
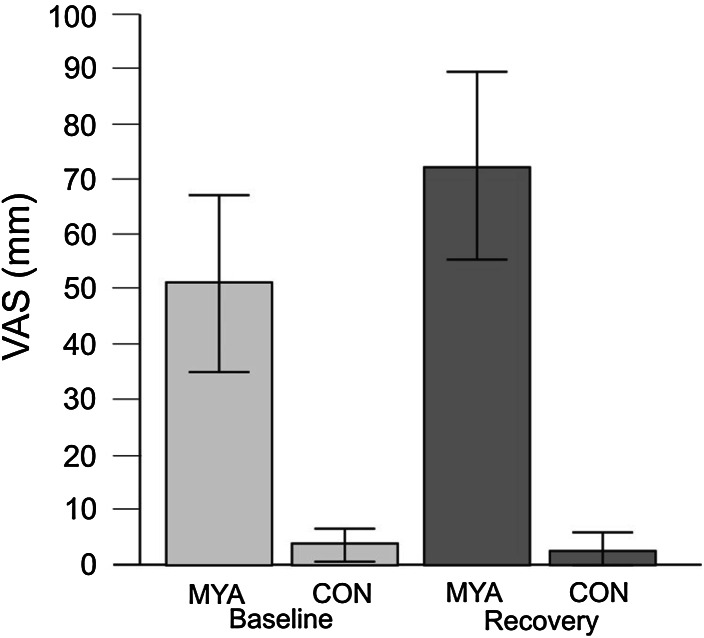
Mean pain intensity in millimetres on a visual analogue scale (VAS; mm) for subjects with chronic myalgia (MYA) and controls (CON). The selection of subjects is based on the pain intensity at recovery (mean shown by the *dark grey bars*). Mean ratings at baseline are shown by the *light grey bars*. *Error bars* represent the standard deviation

Subjects included in this study were from a previous study in our laboratory that investigated serotonin levels in MD samples from female subjects with trapezius myalgia and healthy subjects (Ghafouri et al. 2010; Sjörs et al. 2009). Eleven subjects with trapezius myalgia (MYA) who had rated highest pain intensity at the recovery period (see below) and ten healthy subjects (CON) were selected for the present study (Fig. 1). The selection of MYA with highest pain intensity was done to improve the contrasts between the two groups of subjects in this original study aimed at investigating the interstitium of the trapezius (with and without myalgia) using metabolomics.

Anthropometrics for MYA were mean age 40 years, mean height 165 cm and mean weight 68 kg, and for CON were mean age 42 years, mean height 168 cm, and mean weight 68 kg. The mean body mass indexes (BMI) were 24.0 (SD ± 4.0) and 24.9 (SD ± 3.0) for MYA and CON, respectively. The MYA group was diagnosed with neck pain, tightness of the trapezius muscle (i.e. a feeling of stiffness in the descending region of the trapezius muscle reported by the subject at examination of lateral flexion of the head), palpable tender points of the muscle and normal or slightly decreased range of motion of the cervical column. A clinical examination protocol allowed the examiner to identify and exclude subjects with pain in the trapezius region likely referred from painful tendons or nerve compressions in the neck shoulder area. The median chronic pain duration in MYA was 105 months (range 36–273 months).

All patients participated voluntarily after informed consent. The study was approved by the ethical committee of Linköping University (Dnr M46-07).

### General experimental procedure

The experiment was performed 3–4 weeks after clinical examination and interview. The participants were asked not to use any medications except for paracetamol preparations 3 days before the day of the experiment, and to refrain from intake of caffeine and nicotine 12 h prior to the examination. The subjects were allowed to use paracetamol-based analgesics, since their potential impact on metabolism is considered to be weak; further, the suggested mechanism of paracetamol concerns more central pain mechanism in combination with a weak anti-inflammatory effect (Graham et al. 2013). Participants were also asked not to perform any shoulder or neck-straining exercises 48 h before the study, except for ordinary daily work and/or leisure activities. The participants reported to the laboratory in the morning. They had finished breakfast 1–2 h before the start of the experiment.

### Microdialysis procedure

Two MD catheters (5 and 3,000 kDa cut off) were inserted into the pars descendens of the trapezius muscle on the most painful side for the MYA group (in all patients also the dominant side) and on the dominant side for the CON group. The site for catheter insertion was at a standardised location for all subjects. The insertion point was located at the center of the descending part of the trapezius muscle, midway between the processus spinosus of the seventh cervical vertebra and the lateral end of acromion. The skin and the subcutaneous tissues at the catheters’ entrance and exit sites were anaesthetised with a local injection (0.2–0.5 ml) of Xylocaine (20 mg/ml) without adrenaline, and care was taken not to anaesthetise the underlying muscle. The distance between the entrance and exit sites of the catheters in the skin was approximately 7 cm with at least 5 cm of the catheter in the trapezius muscle ensuring that the entire 30 mm membrane was situated within the muscle tissue. The MD catheters were placed in parallel to the muscle fibres with an inter-catheter distance of approximately 2 cm between the MD catheters. To facilitate the positioning of the MD catheters, the skin at the entrance point was cautiously punctured with a needle, which eliminated the toughness of the skin. Thus, a high control of the tip of the spinal needle was enabled as well as the possibility to slowly pass through skin, subcutaneous tissue and fascia and enter the muscle with the spinal needle. Typically, a brief involuntary contraction and change of resistance were perceived when the tip of the spinal needle entered the fascia and muscle. When the spinal needle was in the correct place, the inner steering pin was removed and the MD catheter was inserted, after which the needle was carefully removed. In this study, the microdialysate from the 3,000 kDa catheter was used. The MD catheters were perfused with a high-precision syringe pump (CMA 107; Carnegie Medicine, Solna, Sweden) at a rate of 5 μl/min with a Ringer acetate solution (Pharmacia & Upjohn, Copenhagen, Denmark) containing 3 mM glucose and 0.5 mM lactate to minimise the risk of draining the interstitial space (Lönnroth et al. 1987).

After catheter insertion, the participants rested 120-min to obtain steady state (Rosendal et al. 2004a; Ernberg et al. 1999), to allow the tissue to recover from possible changes in the interstitial environment induced by the catheter insertion (trauma phase). Although no control molecules for tissue damages were measured, previous studies have shown that a 120 min rest period after the insertion is sufficient to allow the levels of inflammatory substances to return to pre-insertion levels (Flodgren et al. 2005). During the trauma phase, the subjects were given a standardised meal that was served 80 min after start of the experiment. The trauma phase was followed by a 20 min baseline period, still at rest, where the subjects were instructed to sit comfortably in an armchair and rest the neck/shoulder muscles. After this period the subjects preformed a standardised repetitive arm work for 1 h and 40 min. Immediately after the work period, a stress test was implemented. The experiment ended with 80 min of recovery during which the subjects rested. MD samples were collected every 20 min. Samples from the baseline period and the recovery period (pooled for the 60 and 80 min after work time points) were analysed in this study. For the subjects of both MYA and CON, the catheters worked well during the entire experiment. For quality control, each vial (for a particular sample) was weighed before and immediately after sampling to confirm that sampling was working according to the set perfusion rate.

### Work and stress test

A standardised repetitive work was performed using two standardised Valpar Component Work Stations (VCWS08 and VCWS204, Valpar Tucson, USA) and one peg-board exercise; these are previously described (Sjörs et al. 2010). The stations were selected to simulate sedentary or assembly work that requires unilateral or bilateral use of upper extremities. The purpose was to exacerbate pain in the MYA group by performing the work predominantly with their painful side. The three work portions were performed at a standardised work pace and alternated in 20-min intervals in the sequence; VCWS08—peg-board—VCWS204—peg-board—VCWS204—peg-board. The repetitive work test was immediately followed by the Trier social stress test (TSST) which is a standardised and well-validated psychosocial stressor (Kirschbaum et al. 1993). The TSST-test protocol consisted of a 10-min preparatory and information period followed by a 5 min speech and 5 min verbal arithmetic task. For all subjects, the TSST took place between 1.30 p.m. and 3.30 p.m. to minimise confounders from diurnal variation in hormone levels. The experimental sessions were scheduled day 1–10 in the menstrual cycle (i.e. the follicular phase).

### Processing of samples

#### Chemicals

The chemicals used for sample preparation were all of analytical grade, except otherwise stated. The stable isotope-labelled internal standard compound (IS) 13C5-proline, 2H4-succinic acid, 13C5, 15 *N*-glutamic acid, 1,2,3-13C3 myristic acid, 2H7-cholesterol and 13C4 disodium alpha-ketoglutarate were purchased from Cambridge Isotope Laboratories (Andover, MA, USA); 13C12-sucrose, 13C4-palmitric acid and 2H4-butanediamine.2HCl were from Campro (Veenendaal, The Netherlands); 13C6-glucose was from Aldrich (Steinheim, Germany) and 2H6-salicylic acid was from Icon (Summit, NJ, USA). Stock solutions of the IS were prepared either in purified and deionised water (Milli-Q, Millipore, Billerica, MA, USA) or in Methanol (J.T.Baker, Deventer, Holland) at the same concentration 0.5 microgram per microlitre. Metyl stearate was purchased from Sigma (St. Louis, MO, USA). *N*-Metyl-*N*-trimethylsilyltriflouroacetamide (MSTFA) with 1 % trimethylchlorosilane (TMCS) and pyridine (silylation grade) was purchased from Pierce Chemical Co.; heptane was purchased from Fisher Scientific (Loughborough, UK).

#### Sample preparation

Prior to extraction, samples were allowed to thaw at room temperature and then put on ice. Next 225 μl of the extraction buffer (methanol/water 9:1, with 1 l IS each of the concentration 7 ng/μl) was added to 50 μl of the microdialysate. Samples from the recovery period were pooled taking 25 μl from each of the samples from 60 and 80 min after work and TSST. The mixtures were vortexed for approximately 10 s and extracted in a bead mill (MM301 vibration mill, Retsch GmbH & Co. KG, Haan, Germany) at 30 Hz for 2 min. After 2 h at 4 °C on ice, samples were centrifuged at 14,000 rpm for 10 min at 4 °C. A 100-μl aliquot was transferred to a GC vial and evaporated using a speedvac to dryness. Methoxymation with 30 μl methoxyamine solution in pyridine (15 μg/μl) was carried out in room temperature for 1 h, after which 30 μl of heptanes (with 0.5 μg of methyl stearate as injection IS) was added (Trygg et al. 2005; Wibom et al. 2010).

#### GC–MS

A 1-μl aliquot of derivatised sample was injected in splitless mode by Agilent 7683 Series autosampler (Aligent, Atlanta, GA, USA) into an Aligent 6980 GC equipped with a 10 m × 0.18 mm i.d. fused-silica capillary column chemically bonded with 0.18 μm DB5-MS stationary phase (J&W Scientific, Folsom, CA, USA). The injector temperature was 270 °C. The carrier gas was helium, set at a constant flow of 1 mL/min through the purge flow rate of 20 ml/min and an equilibrant time of 1 min. The column temperature was initially kept at 70 °C for 2 min and then increased from 70 to 320 °C at 30 °C/min, where it was kept for 2 min. The effluent from the column was introduced to the ion source of Pegasus III TOFMS (Leco Corp., St Joseph, MI). The transfer temperature was set at 250 °C and the ion source temperature at 200 °C. Ions were generated by a 70 eV electron beam at a current of 2.0 mA. Masses were acquired from *m/z* 50 to 800 at a rate of 30 spectra per second, and the acceleration voltage was turned on after a solvent delay of 165 s. Retention indexes were calculated from the retention times obtained from the injection of a homologous series of n-alkanes (C_12_–C_32_) for each batch. All samples were run in randomised order (Wibom et al. 2010).

#### Hierarchical multivariate curve resolution

Analysing complex samples gives rise to overlapping peaks in the GC chromatograms and thus mixed mass spectra from the TOF–MS analysis. To solve this problem, a multivariate curve resolution method named hierarchical multivariate curve resolution (H-MCR) has been applied. In short, the H-MCR generates a matrix where all MD samples are described by a common set of variables, each representing one metabolite (Jonsson et al. 2005). The data can then be subjected to multivariate data analysis as presented below. A recently implemented internal validation step in the H-MCR algorithm assures extraction of robust and reliable metabolite profiles (Thysell et al. 2012). Together GC–MS and H-MCR constitute a reproducible platform that allows for generation of global metabolomics for multiple sample comparisons within and between sample batches (Jonsson et al. 2006; Thysell et al. 2012).

### Statistics

Data from samples collected from patients with chronic trapezius myalgia were compared to the group of healthy subjects during either baseline or recovery.

#### Systematic differences—orthogonal partial least-squares

To analyse for systematic differences in metabolite content, the metabolite data were modelled and interpreted for the complex interactions acquired in this study using orthogonal partial least-squares with discriminant analysis (OPLS-DA) (Trygg and Wold 2002). In data-sets from GC–TOF–MS analysis of metabolites, the number of variables (metabolites) greatly exceeds the number of observation and the variables are often highly correlated. The multivariate projection method OPLS-DA used in this study has the advantage of being able to deal with such data and also to separate the variability among the metabolites that is directly related to group separation from variability not related to group separation.

In more detail, OPLS-DA is a supervised multivariate data projection method used to relate a set of predictor variables (*X* or metabolites in this study) to a response matrix (*Y*) that represents predefined sample classes (myalgic or healthy). This method can then be used to predict class identity and to extract specific features among the predictor variables (metabolites) distinguishing between the predefined sample classes. OPLS operates by dividing the systematic variation in *X* into two parts: one part is the linearity related to *Y*, and thus can be used to predict *Y*, and one part is uncorrelated (i.e. orthogonal) to *Y*. In this process, each variable in *X* is associated with a weight w* for each model component, which represents the variable’s covariation with *Y* in that component. Also, each variable is assigned an OPLS-DA variable importance in the projection (VIP) value. Since VIP accounts for the variable importance using all model components (predictive and orthogonal), in models with more than one component, VIP does not directly relate to the predictive component in the model. In this study, a VIP value above 1.0, in combination with w* for the predictive component, was used to highlight significant metabolites or metabolite patterns. Metabolites which contributed to the explanation of group affiliation are presented in Table 1.

**Table 1:** All identified metabolites with OPLS loadings value (w*) and variable of importance in the projection (VIP) value. Principal abundance of the metabolite in controls (CON) and patients experiencing trapezius myalgia (MYA); where negative loading values (w*) refers to a higher occurrence in MYA and positive loadings refers to a higher occurrence in CON. Metabolites with w* > 0.1 are given in bold. Metabolites were tested for their significance according to Mann–Whitney *U* test and are given an * in the table if considered significant (*p* > 0.05)

ID	Metabolite	w*(MYA/CON)	VIP
Amino acids			
1	l-Alanine	−0.028	0.366
16	l-Valine	0.019	0.251
22	l-Leucine	−0.021	0.281
26	Isoleucine	0.034	0.444
27	l-Proline	0.091	1.196
28	Glycine	0.031	0.407
37	Glutamic acid	0.011	0.150
38	l-serine	0.028	0.363
43	Glutamic acid	0.033	0.435
46	Threonine. DL-	−0.043	0.567
59	**l** **-Glutamine**	0.106	0.373
68	l-Glutamic acid	0.085	1.123
69	Pyroglutamic acid	0.010	0.135
80	Glutamine	−0.021	0.279
81	l-Tyrosine	0.046	0.607
86	Ornithine	−0.022	0.286
87	l-Glutamic acid	0.073	0.958
88	Phenylalanine	0.052	0.678
98	Putrescine*	0.177	2.327
99	Putrescine	0.068	0.889
106	Arginine	−0.027	0.358
116	l-Lysine	0.011	0.150
117	Tyrosine	−0.018	0.239
118	Tyrosine	−0.044	0.582
141	Phenylalanine	−0.017	0.220
146	l-Cystine	−0.059	0.777
Carbohydrates			
31	Glyceric acid	0.020	0.264
85	d-Ribofuranose	0.040	0.525
91	Zylose	0.056	0.738
95	Xylose	0.035	0.462
96	1.6-anhydro-beta-d-Glucose	−0.013	0.171
97	**Arabitol**	0.101	1.334
104	Arabitol	0.009	0.113
107	Glucose	−0.093	1.229
112	**Glucose**	0.140	1.841
113	Galactose	−0.028	0.363
114	Glucose	0.098	1.291
115	Glucose	−0.005	0.064
120	Myo-Inositol	−0.087	1.144
125	**Beta-** d -Glucopyranose*	−0.164	2.156
129	Beta-d-Methylglucopyranoside	−0.046	0.608
135	Myo-inositol	0.011	0.145
156	Glucose	0.087	1.151
157	Maltose	0.036	0.482
172	Maltotriose	0.014	0.184
Fatty acids			
6	Butanoic acid	−0.065	0.852
9	2-Aminobutyric acid	−0.035	0.467
23	**Glycerol**	0.123	1.614
94	Lauric acid	0.033	0.435
105	Glycerol	0.077	1.012
109	Myristic acid*	0.163	2.140
110	**Myristic acid**	0.101	1.332
111	Myristic acid	0.038	0.498
130	Palmitic acid	0.079	1.040
133	Palmitic acid, trimethylsilyl ester	0.008	0.101
137	**Stearic acid methyl ester**	0.103	1.361
Other			
11	Urea	−0.037	0.491
21	Urea	−0.085	1.121
24	Phosphoric acid	−0.003	0.036
29	Succinic acid	0.068	0.894
47	Lactic acid	−0.056	0.738
53	2.5-Diaminovalerolactam	−0.028	0.373
64	Salicylic acid	0.048	0.626
65	Salicylic acid	0.028	0.374
66	**Salicylic acid**	0.195	2.566
67	Salicylic acid	0.002	0.0280
73	Creatinine	−0.039	0.517
75	**Alpha-Ketoglutarate**	0.127	1.672
76	Alpha-Ketoglutarate	0.015	0.201
124	d-Galactono-1.4-lactone	−0.097	1.281
136	Uric acid	0.036	0.477
153	Pyruvic acid	0.039	0.516

#### Data pretreatment and analysis strategies

The data matrix (*X*) produced by H-MCR was scaled to unit variance and screened for inconsistencies, using both principal component analysis (PCA) (Trygg and Wold 2002) and OPLS-DA.

#### Single metabolite analysis

Prior to the analysis, the data were tested for normal distribution using Kolmagorov–Smirnov resulting in all metabolites being analysed using non-parametric Mann–Whitney *U* test. The two-tailed significance level was set to *α* = 0.05.

## Results

The metabolomics approach could identify 74 metabolites in the interstitium fluid of human trapezius muscle. The majority of metabolites belonged to amino acids, fatty acids and carbohydrates.

### Baseline

Microdialysate from healthy (CON) and myalgic (MYA) trapezius muscle was analysed for metabolite content; two of the controls were excluded due to the insufficient amount of dialysate at baseline. The OPLS-DA of group affiliation (MYA or CON) using the metabolites as regressors did not generate an explanatory model. According to the Mann–Whitney *U* test, two metabolites differed significantly, with higher abundances in the interstitium of the MYA muscle: l-leucine (*p* = 0.041) and pyroglutamic acid (*p* = 0.034).

### Recovery

**Fig. 2:**
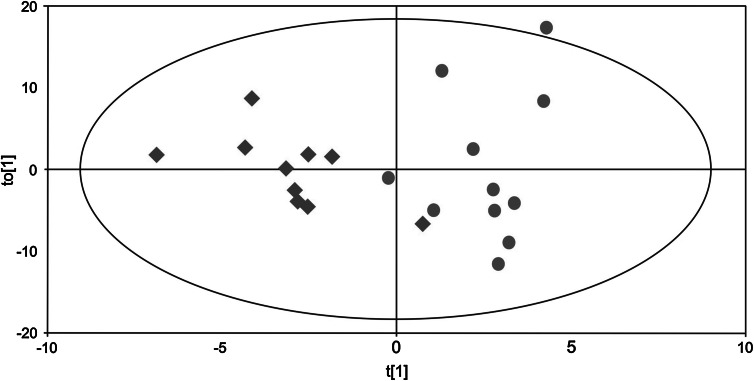
OPLS-DA model of controls (CON) and patients with chronic trapezius myalgia (MYA) at recovery. Hence, the *plot* describes the multivariate relationships between subjects and groups. *Red diamonds* represent CON and *green circles* MYA. (*R* ^2^(cum) = 0.532 and *Q* ^2^ = 0.295, *R* ^2^ predictive component = 0.071 and *R* ^2^ two orthogonal components 0.461). *t*[1] is the score value of the predictive component and to[1] is the score value of the first orthogonal component

**Fig. 3:**
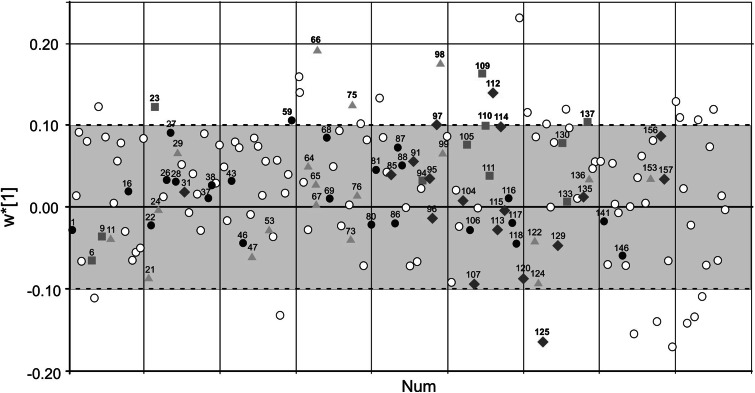
OPLS-DA loadings plot of the analysed metabolites: fatty acids, carbohydrates, proteins and other metabolites. *Black dots* represent protein metabolites, *orange squares* fatty acids and *green diamonds* carbohydrates and *blue triangles* other metabolites: Metabolites more abundant in MYA (VIP > 1 and w* > 0.1) and metabolites more abundant in CON (VIP > 1 and w* < −0.1) are visualised here outside the *grey area* and listed in Table 1. *Round unfilled* circles represent unidentified metabolites

It was possible to distinguish the two groups of subjects based on systematic difference in metabolites using OPLS-DA (Model characteristics: *R* ^2^ = 0.532 and *Q* ^2^ = 0.295) (Fig. 2). In Fig. 2, the separation of the two groups is visualised in the OPLS-DA score plot. This metabolomics approach allowed us to identify a number of metabolites that in the multivariate context differed in abundance between MYA and CON. Figure 3 shows metabolites plotted according to their loading weight value (w*) and including their identification number. The detected metabolites together with the metabolites of interest are listed in Table 1.

The metabolite content in the muscle interstitium differed between CON and MYA with respect to the abundance of amino acids, fatty acids, carbohydrates and other metabolites (Table 1). In MYA, there was an increased abundance of amino acids (sorted in descending order according to w*): Putrescine, l-Glutamine, l-Proline, and l-Glutamic acid; carbohydrates: Glucose and Arabitol; fatty acids: Myristic acid, Glycerol, Stearic acid, and Palmitic acid. In CON, there was an increased abundance of carbohydrates: beta-d-Glucopyranose, d-Galactono-1.4-lactone, Glucose, and Myo-Inositol. Other metabolites of interest were: Alpha-Ketoglutarate—more abundant in the interstitium of MYA, and Urea—more abundant in the interstitium of CON. Some metabolites were detected several times as in Table 1. According to Mann–Whitney *U* tests two metabolites were significantly more abundant in the interstitium of MYA—myristic acid (*p* = 0.043) and putrescine (*p* = 0.013), while in the interstitium of CON glucopyranose (*p* = 0.006) was significantly more abundant.

## Discussion

This study presents a novel approach in using metabolomics to analyse microdialysates collected from myalgic and healthy muscles. Microdialysates were collected from extracellular space in the trapezius muscle before (baseline) and after (recovery) exposure to a standardised work and stress test. Systematic differences in the abundance of metabolite content were visualised in multivariate models when many possibly inter-related metabolites in the samples differed between the groups. To analyse for differences in content of individual metabolites, the Mann–Whitney *U* test was applied.

The extracellular content of metabolites reflects intracellular processes of the muscle or other adjacent cells and may also be consequences of systemic processes. Previous metabolomic studies regarding muscle activity and metabolism have foremost been performed in exercise studies (Yan et al. 2009; Chorell et al. 2009, 2012; Pohjanen et al. 2007).

### Baseline

Pyroglutamic acid and l-Leucine were the two metabolites with a significantly increased abundance in the MYA at baseline compared to CON. Pyroglutamic acid is a ring-like formation of glutamate (Katchalsky and Paecht 1954) and is considered an intermediate acting as a reservoir for glutamate (Abraham and Podell 1981). Glutamate is a peripheral and central neurotransmitter involved in different aspects of pain, e.g. long-term potentiation (reviewed by Miller et al. 2011). Glutamate is released from peripheral afferent nerve terminals (Svensson et al. 2003) and receptors are located on the peripheral ends of small-diameter primary afferents. When glutamate is released into the synaptic cleft, nociceptors are activated (Miller et al. 2011; Coggeshall and Carlton 1998). As visualised in Table 1, MYA rated higher pain in the trapezius compared to CON. Two previous studies showed an increased abundance of glutamate in the interstitium of MYA (Rosendal et al. 2004b; Larsson et al. 2008) which may be in coherence with our findings. In the OPLS-DA model concerning recovery, α-ketoglutarate was a metabolite of interest more abundant in the interstitium of MYA (Table 1). In addition, the synthesis of α-Ketoglutarate, a key intermediate in the TCA cycle, occurs by transamination of glutamate or through the action of glutamate dehydrogenase on glutamate. Other amino acids related to glutamate, l-glutamine and l-glutamic acid were also more abundant in the MYA group at recovery.

The role of l-leucine in muscle remodelling in myalgic muscle has, to our knowledge, not been investigated. However, in a study by Mackey et al., an increased number of satellite cells was found in the myalgic trapezius muscle indicating a greater potential or need for remodelling (Mackey et al. 2010), which is in agreement with the higher abundance of l-Leucine in the present study. l-Leucine is an essential amino acid (EAA) shown to remodel human skeletal muscle through phosphorylation of the mammalian target of rapamycin (mTOR) and the subsequent phosphorylation of the 70 kD S6 protein kinase (Greiwe et al. 2001; Blomstrand and Essen-Gustavsson 2009). Also, l-Leucine has been suggested to stimulate glycogen synthesis through activation of glycogen synthase kinase-3 (Peyrollier et al. 2000). High initial concentrations of muscle glycogen stimulate glycogen breakdown (Hespel and Richter 1992). This coincides with a recent finding where higher abundances of glycogen phosphorylase and phosphoglucomutase-1 were shown in myalgic muscle biopsies (Hadrevi et al. 2013).

### Recovery

Previous studies have implied that some interstitial substances in myalgic muscle had not returned to baseline values after 60 min of rest following a low level repetitive work (Sjogaard et al. 2010; Flodgren et al. 2010; Rosendal et al. 2004b). In the present study, the recovery sample was collected as a pooled sample 60 and 80 min at the end of the work and stress test. In agreement with earlier studies on single metabolites (Sjogaard et al. 2010; Flodgren et al. 2010), differences in the metabolite pattern between healthy and myalgic muscles were visualised in the explanatory OPLS-DA models at recovery (Figs. 2, 3). This systematic difference in metabolite content suggests an altered metabolite patterns during recovery after the work and stress provocation. The differences in metabolite content between groups during recovery but not at baseline may indicate a need for a sufficient provocation of the muscular system to detect systematic effects in a relatively limited number of subjects.

#### Fatty acids and carbohydrates

According to the OPLS-DA model glycerol, palmitic acid (hexadecanoic acid), stearic acid (octadecanoic acid) and myristic acid (tetradecanoic acid) were more abundant in the extracellular compartments of MYA (Table 1). In addition, myristic acid (tetradecanoic acid) was significant also in the univariate test.

Glycerol found in the interstitium of the muscle is theoretically produced by the hydrolysis of triglycerides of muscle cells, plasma lipoproteins and adipocytes (Sjostrand et al. 2002). Increased levels of muscle glycerol have been shown in contracting muscle from patients with lactate dehydrogenase deficiency (Kanno and Maekawa 1995), in working leg muscle (van Hall et al. 2002) and during low exercise intensity (Stallknecht et al. 2004).

Stearic acid and palmitic acid are substrates for synthesis of the bioactive lipid molecules related to pain sensations; *N*-Palmitoylethanolamide (PEA) and *N*-stearolethanolamide (SEA). SEA is endocannabinoid-like fatty acids that induces analgesic effects (Maccarrone et al. 2002). PEA has been reported as an anti-inflammatory and anti-nociceptive agent (Calignano et al. 2001; Darmani et al. 2005). Elevated levels of these substances have recently been found in myalgic women compared to healthy controls (Ghafouri et al. 2011).

One of the suggested contributing mechanisms to trapezius myalgia is stress (Lundberg et al. 1999; Knardahl 2002). Also, patients suffering from chronic trapezius myalgia are exposed to physiological stress due to sustained pain. During acute physiological stress, the metabolism is altered, as an increase in the breakdown of fat (the lipolysis) due to adrenergic mechanisms (Hagstrom-Toft et al. 1993). An increased drive of the sympathetic nervous system (SNS) also facilitates lipid mobilisation (Coppack et al. 1994; Bickerton et al. 2008). Though, after repeated adrenalin treatment, a suppression of the basal and adrenaline-stimulated lipolysis has been observed (Townsend et al. 1994). Also, in vitro studies have shown that targets cells become desensitised after sustained changes in SNS activity or plasma catecholamine levels, as in chronic stress situations (Lafontan and Berlan 1993). The excess amount of fatty acids abundant in the interstitium of MYA during the recovery phase might fit with the explanation that there is a desensitisation of the target cells (the muscle cells) due to the physiological adaptations to prolonged stress (Townsend et al. 1994). Additionally, studies made on patients with chronic pain conditions show an increased intramuscular fat content, i.e. between the muscle cells (Gerdle et al. 2013a; Elliott et al. 1976). Also, a recent study shows down-regulated mRNA content of pyruvate dehydrogenase (PDH-E1α) (Sjøgaard et al. 2013). A possible explanation to this could be an increased fatty acid metabolism providing the citric acid cycle with Acetyl-CoA. Carbohydrate metabolism is also affected by chronic stress, although no such conclusion could be made from the results presented here, since in the OPLS-DA model glucose metabolites did appear in both MYA and CON. Also, glucopyranose which is a cyclised sugar metabolite was significantly more abundant in CON according to the Mann–Whitney *U* test.

#### Amino acids

Putrescine, l-ornithine, l-arginine and spermidine (not detected in this analysis) are polyamine amino acids. Putrescine was significantly more abundant in MYA at recovery according to the univariate test. Putrescine is synthesised from l-arginine via l-ornithine (Pegg 2009) and is transported in and out over cell membranes energy-dependant (Pegg 1988; Seiler and Dezeure 1990). It is an extracellular marker of muscle turnover and an indicator of muscle growth rate (Lee and MacLean 2011). Increased polyamine levels in skeletal muscle have previously been found in patients suffering from Duchenne muscle dystrophy (Kaminska et al. 1981), which is associated with high muscle turnover and connective tissue proliferation (Kaminska et al. 1981). Also limb girdle patients with enlarged and split muscle fibres with widespread regeneration and degeneration express higher levels of polyamines (Kaminska et al. 1981; Rudman et al. 1980). Hence, an increased abundance of polyamines is indicative of a higher muscle turnover in myalgic patients compared with healthy. Interestingly, Mackey et al. recently reported that type 1 muscle fibres in the trapezius of subjects with chronic myalgia have higher density of satellite cells, and myonuclei gives support to increased myogenic activity (Mackey et al. 2010).

### Experimental design

Pyruvate and lactate have previously been considered biomarkers of chronic myalgia (Gerdle and Larsson 2012). In the present study, neither lactate nor pyruvate occurred as of interest in the OPLS-DA model or the additional univariate analysis at baseline. At recovery, the metabolites were detected in the analysis but did not appear as of interest (Table 1). One possibility is that lactate was, in fact, elevated in myalgic muscles at the end of the work period but had time to normalise during the stress test, when muscle activity was low. Another possibility is that the provocation of the muscle was not large enough in this experiment. In the previously presented studies, there were different experimental settings when significant changes in pyruvate and lactate concentrations at baseline were detected. The work stations in the present study were chosen to mimic low-load repetitive work, similar to industrial assembly work, with an intensity that was tolerable to the patients for relatively long time. The PEGs and handles used in other repetitive work simulations have had a higher weight, 100 g (Sjogaard et al. 2010) and 130 g (Flodgren et al. 2010) compared to the PEGs used in this study with a 10 g weight. In reality, the weight experienced by the subject was greater than 10 g considering also the weight of the arm; this applies, however, to all studies of this type. Studies using 23 g weights (Rosendal et al. 2004a, b) did not obtain increased lactate and pyruvate concentrations in the recovery phase which is in accordance with the results presented here. Thus, higher loads are possibly needed to elicit such metabolic changes and lactate and pyruvate might be markers of the myalgic muscles response to work load rather than pain sensations. But, on the other hand, several studies of chronic trapezius myalgia have reported significantly increased concentrations of these metabolites at baseline (Rosendal et al. 2004a, b). Changes in metabolites between healthy and myalgic muscle in the recovery phase could potentially be due to differences in blood flow clearance of metabolites from the muscle. However, altered in blood flow has been foremost observed during exercise or mental tasks. The samples in our study were collected 60–80 min at the end of the intervention, when there were no differences in potential hemodynamic parameters (Elcadi et al. 2013).

Moreover, systematic alterations in metabolites were found according to the OPLS-DA model, implying that the metabolism is still affected by the previous work and stress intervention. Another explanation was that the patients with pain had a lower severity than in earlier studies due to the fact that the procedures with potential to induce increased pain intensity were relatively extensive, which in turn may have created a selection of patients.

Pain intensity (Fig. 1) during the work and stress test was higher in MYA compared CON, and the work test did elicit a significant increase in pain in the patient group (Sjörs et al. 2009), which is an important factor separating groups and attributing the present study as a pain and stress study.

### Methodological considerations

The detection of metabolites is in many ways dependent on dietary intake. In this study, a fixed meal was served during the experiments which would help control for dietary metabolites; however, it is possible that metabolites from diet could linger in the system for longer times which can compromise the analysis.

Also, the physical activity patterns, per se, for whole body or only regarding the trapezius muscle can differ between patients and controls (Andersen et al. 2008a, b). However, strength and/or endurance were not measured so we cannot be sure of the contribution in the present study. A lower physical activity pattern can also be the consequence of pain, i.e. deconditioning. Physical activity has an effect on, for example, the fatty acid metabolism, as it affects the beta-oxidation (Konig et al. 2003). Studies that, to a large extent, control for work exposure, physical fitness and weekly dietary intake are likely preferred but difficult to achieve. On the other hand, the activity pattern of the trapezius muscle may be higher or more prolonged in MYA than in CON (Sjörs et al. 2009; Peolsson et al. 2008; Larsson et al. 2000a; Elert et al. 1992).

In investigations of metabolic patterns and potential metabolic markers-based projection models, interpretation of what variable is important must be made with caution. VIP > 1 is generally accepted as criteria for selecting a variable (Eriksson et al. 2001). Also, in models with more than one component, VIP might not give the correct relationship between the pattern of variables and response (*Y*). In the case of OPLS-DA loading, weights of the predicative component will always give a correct relationship; however, there are difficulties in defining a threshold based on loading weights [for review Mehmood et al. (2012)]. Thus, in the present study, a combination of VIP and the weights of the predictive component were used.

## Conclusions

Since previous studies analysing metabolite content in myalgic muscle have focused only on a few metabolites, e.g. lactate, pyruvate, glutamate and glucose, the present study serves as a complement elucidating the possible systematic changes in metabolites. Previous comparisons between healthy and myalgic muscle during low-load repetitive work show that low-intensity activation evokes no prolonged change in single metabolite content in the myalgic muscle (Rosendal et al. 2004a, b). However, in our study, changes in single metabolites during baseline were detected. Also, single metabolite and systematic changes after the low intensity work and stress tests were found in the muscle interstitium. The metabolomics technique enables a comprehensive analysis of the muscle microdialysate. The physiological effects after stress and pain induced low-load work were detected showing systematic changes in metabolites.

## References

[CR1] Abraham GN, Podell DN (1981). Pyroglutamic acid. Non-metabolic formation, function in proteins and peptides, and characteristics of the enzymes effecting its removal. Mol Cell Biochem.

[CR2] Andersen LL, Holtermann A, Jorgensen MB, Sjogaard G (2008). Rapid muscle activation and force capacity in conditions of chronic musculoskeletal pain. Clin Biomech (Bristol, Avon).

[CR3] Andersen LL, Nielsen PK, Sogaard K, Andersen CH, Skotte J, Sjogaard G (2008). Torque-EMG-velocity relationship in female workers with chronic neck muscle pain. J Biomech.

[CR4] Andersen LL, Suetta C, Andersen JL, Kjaer M, Sjogaard G (2008). Increased proportion of megafibers in chronically painful muscles. Pain.

[CR5] Andersen LL, Blangsted AK, Nielsen PK, Hansen L, Vedsted P, Sjogaard G, Sogaard K (2010). Effect of cycling on oxygenation of relaxed neck/shoulder muscles in women with and without chronic pain. Eur J Appl Physiol.

[CR6] Bickerton AS, Roberts R, Fielding BA, Tornqvist H, Blaak EE, Wagenmakers AJ, Gilbert M, Humphreys SM, Karpe F, Frayn KN (2008). Adipose tissue fatty acid metabolism in insulin-resistant men. Diabetologia.

[CR7] Blomstrand E, Essen-Gustavsson B (2009). Changes in amino acid concentration in plasma and type I and type II fibres during resistance exercise and recovery in human subjects. Amino Acids.

[CR8] Calignano A, La Rana G, Piomelli D (2001). Antinociceptive activity of the endogenous fatty acid amide, palmitylethanolamide. Eur J Pharmacol.

[CR9] Chorell E, Moritz T, Branth S, Antti H, Svensson MB (2009). Predictive metabolomics evaluation of nutrition-modulated metabolic stress responses in human blood serum during the early recovery phase of strenuous physical exercise. J Proteome Res.

[CR10] Chorell E, Svensson MB, Moritz T, Antti H (2012). Physical fitness level is reflected by alterations in the human plasma metabolome. Mol BioSyst.

[CR11] Coggeshall RE, Carlton SM (1998). Ultrastructural analysis of NMDA, AMPA, and kainate receptors on unmyelinated and myelinated axons in the periphery. J Comp Neurol.

[CR12] Coppack SW, Jensen MD, Miles JM (1994). In vivo regulation of lipolysis in humans. J Lipid Res.

[CR13] Darmani NA, Izzo AA, Degenhardt B, Valenti M, Scaglione G, Capasso R, Sorrentini I, Di Marzo V (2005). Involvement of the cannabimimetic compound, *N-*palmitoyl-ethanolamine, in inflammatory and neuropathic conditions: review of the available pre-clinical data, and first human studies. Neuropharmacol.

[CR14] Dunn WB, Ellis DI (2005). Metabolomics: current analytical platforms and methodologies. TrAC Trends Anal Chem.

[CR15] Elcadi GH, Forsman M, Aasa U, Fahlstrom M, Crenshaw AG (2013). Shoulder and forearm oxygenation and myoelectric activity in patients with work-related muscle pain and healthy subjects. Eur J Appl Physiol.

[CR16] Elert JE, Rantapaa-Dahlqvist SB, Henriksson-Larsen K, Lorentzon R, Gerdle BU (1992). Muscle performance, electromyography and fibre type composition in fibromyalgia and work-related myalgia. Scand J Rheumatol.

[CR17] Elliott JM, O’Leary S, Sterling M, Hendrikz J, Pedler A, Jull G (1976). Magnetic resonance imaging findings of fatty infiltrate in the cervical flexors in chronic whiplash. Spine.

[CR18] Eriksson L, Johansson E, Kettaneh-Wold N, Wold S (2001) Multi-and megavariate data analysis. Umetrics Academy, Umeå, Sweden

[CR19] Ernberg M, Hedenberg-Magnusson B, Alstergren P, Kopp S (1999). The level of serotonin in the superficial masseter muscle in relation to local pain and allodynia. Life Sci.

[CR20] Flodgren GM, Crenshaw AG, Alfredson H, Fahlstrom M, Hellstrom FB, Bronemo L, Djupsjöbacka M (2005). Glutamate and prostaglandin E(2) in the trapezius muscle of female subjects with chronic muscle pain and controls determined by microdialysis. Eur J Pain.

[CR21] Flodgren G, Crenshaw AG, Hellström F, Fahlström M (2010). Combining microdialysis and near-infrared spectroscopy for studying effects of low-load repetitive work on the intramuscular chemistry in trapezius myalgia. J Biomed Biotechnol.

[CR22] Gerdle B, Larsson B (2012). Potential muscle biomarkers of chronic myalgia in humans—a systematic review of microdialysis studies. Biomarker Chap.

[CR23] Gerdle B, Forsgren M, Bengtsson A, Leinhard OD, Sören B, Karlsson A, Brandejsky V, Lund E, Lundberg P (2013). Decreased muscle concentrations of ATP and PCR in the quadriceps muscle of fibromyalgia patients—a 31P MRS study. Eur J Pain.

[CR24] Gerdle B, Larsson B, Forsberg F, Ghafouri N, Karlsson L, Stensson N, Ghafouri B (2013). Chronic Widespread Pain: increased Glutamate and lactate concentrations in the trapezius muscle and plasma. Clin J Pain.

[CR25] Ghafouri B, Larsson BK, Sjors A, Leandersson P, Gerdle BU (2010). Interstitial concentration of serotonin is increased in myalgic human trapezius muscle during rest, repetitive work and mental stress—an in vivo microdialysis study. Scand J Clin Lab Invest.

[CR26] Ghafouri N, Ghafouri B, Larsson B, Turkina MV, Karlsson L, Fowler CJ, Gerdle B (2011). High levels of *N*-palmitoylethanolamide and *N*-stearoylethanolamide in microdialysate samples from myalgic trapezius muscle in women. PLoS One.

[CR27] Graham GG, Davies MJ, Day RO, Mohamudally A, Scott KF (2013). The modern pharmacology of paracetamol: therapeutic actions, mechanism of action, metabolism, toxicity and recent pharmacological findings. Inflammopharmacology.

[CR28] Greiwe JS, Kwon G, McDaniel ML, Semenkovich CF (2001). Leucine and insulin activate p70 S6 kinase through different pathways in human skeletal muscle. Am J Physiol Endocrinol Metab.

[CR29] Hadrevi J, Ghafouri B, Larsson B, Gerdle B, Hellström F (2013). Multivariate modeling of proteins related to trapezius myalgia, a comparative study of female cleaners with or without pain. PLOS one.

[CR30] Hagstrom-Toft E, Arner P, Wahrenberg H, Wennlund A, Ungerstedt U, Bolinder J (1993). Adrenergic regulation of human adipose tissue metabolism in situ during mental stress. J Clin Endocrinol Metab.

[CR31] Hespel P, Richter EA (1992). Mechanism linking glycogen concentration and glycogenolytic rate in perfused contracting rat skeletal muscle. Biochem J.

[CR32] Jonsson P, Sjövik-Johansson E, Wuolikainen A, Lindberg J, Schuppe-Koistinen I, Kusano M, Sjöström M, Trygg J, Moritz T, Antti H (2006). Predictive metabolite profiling applying hierarchical multivariate curve resolution to GC/MS data—a potential tool for multi-parametric diagnosis. J Proteome Res.

[CR33] Kadi F, Hägg G, Håkansson R, Holmner S, Butler-Browne GS, Thornell LE (1998). Structural changes in male trapezius muscle with work-related myalgia. Acta Neuropathologica.

[CR34] Kadi F, Waling K, Ahlgren C, Sundelin G, Holmner S, Butler-Browne GS, Thornell LE (1998). Pathological mechanisms implicated in localized female trapezius myalgia. Pain.

[CR35] Kaminska AM, Stern LZ, Russell DH (1981). Altered muscle polyamine levels in human neuromuscular diseases. Ann Neurol.

[CR36] Kanno T, Maekawa M (1995). Lactate dehydrogenase M-subunit deficiencies: clinical features, metabolic background, and genetic heterogeneities. Muscle Nerve.

[CR37] Katchalsky A, Paecht M (1954). Phosphate anhydrides of amino acids. J Am Chem Soc.

[CR38] Kirschbaum C, Pirke KM, Hellhammer DH (1993). The ‘Trier Social Stress Test’–a tool for investigating psychobiological stress responses in a laboratory setting. Neuropsychobiology.

[CR39] Knardahl S (2002). Psychophysiological mechanisms of pain in computer work: the blood vessel-nociceptor interaction hypothesis. Work Stress.

[CR40] Konig D, Vaisanen SB, Bouchard C, Halle M, Lakka TA, Baumstark MW, Alen M, Berg A, Rauramaa R (2003). Cardiorespiratory fitness modifies the association between dietary fat intake and plasma fatty acids. Eur J Clin Nutr.

[CR41] Lafontan M, Berlan M (1993). Fat cell adrenergic receptors and the control of white and brown fat cell function. J Lipid Res.

[CR42] Larsson S, Bengtsson A, Bodegård L, Henriksson K, Larsson J (1988). Muscle changes in work-related chronic myalgia. Acta Orthop Scand.

[CR43] Larsson B, Bjork J, Elert J, Gerdle B (2000). Mechanical performance and electromyography during repeated maximal isokinetic shoulder forward flexions in female cleaners with and without myalgia of the trapezius muscle and in healthy controls. Eur J Appl Physiol.

[CR44] Larsson B, Bjork J, Henriksson KG, Gerdle B, Lindman R (2000). The prevalences of cytochrome c oxidase negative and superpositive fibres and ragged-red fibres in the trapezius muscle of female cleaners with and without myalgia and of female healthy controls. Pain.

[CR45] Larsson B, Rosendal L, Kristiansen J, Sjogaard G, Sogaard K, Ghafouri B, Abdiu A, Kjaer M, Gerdle B (2008). Responses of algesic and metabolic substances to 8 h of repetitive manual work in myalgic human trapezius muscle. Pain.

[CR46] Lee NK, MacLean HE (2011). Polyamines, androgens, and skeletal muscle hypertrophy. J Cell Physiol.

[CR47] Lindman R, Hagberg M, Ängqvist KA, Söderlund K, Hultman E, Thornell LE (1991). Changes in muscle morphology in chronic trapezius myalgia. Scand J Work Environ Health.

[CR48] Lönnroth P, Jansson P, Smith U (1987). A microdialysis method allowing characterization of intercellular water space in humans. Am J Physiol.

[CR49] Lundberg U, Dohns IE, Melin B, Sandsjo L, Palmerud G, Kadefors R, Ekstrom M, Parr D (1999) Psychophysiological stress responses, muscle tension, and neck and shoulder pain among supermarket cashiers. J Occup Health Psychol 4 10.1037//1076-8998.4.3.245 10431284

[CR50] Maccarrone M, Cartoni A, Parolaro D, Margonelli A, Massi P, Bari M, Battista N, Finazzi-Agro A (2002). Cannabimimetic activity, binding, and degradation of stearoylethanolamide within the mouse central nervous system. Mol Cell Neurosci.

[CR51] Mackey AL, Andersen LL, Frandsen U, Suetta C, Sjogaard G (2010). Distribution of myogenic progenitor cells and myonuclei is altered in women with vs. those without chronically painful trapezius muscle. J Appl Physiol.

[CR52] Mehmood T, Bohlin J, Kristoffersen AB, Saebo S, Warringer J, Snipen L (2012). Exploration of multivariate analysis in microbial coding sequence modeling. BMC Bioinform.

[CR53] Miller KE, Hoffman EM, Sutharshan M, Schechter R (2011). Glutamate pharmacology and metabolism in peripheral primary afferents: physiological and pathophysiological mechanisms. Pharmacol Ther.

[CR54] Pegg AE (1988). Polyamine metabolism and its importance in neoplastic growth and a target for chemotherapy. Cancer Res.

[CR55] Pegg AE (2009). Mammalian polyamine metabolism and function. IUBMB Life.

[CR56] Peolsson M, Larsson B, Brodin LA, Gerdle B (2008). A pilot study using Tissue Velocity Ultrasound Imaging (TVI) to assess muscle activity pattern in patients with chronic trapezius myalgia. BMC Musculoskelet Disord.

[CR57] Peyrollier K, Hajduch E, Blair AS, Hyde R, Hundal HS (2000). l-leucine availability regulates phosphatidylinositol 3-kinase, p70 S6 kinase and glycogen synthase kinase-3 activity in L6 muscle cells: evidence for the involvement of the mammalian target of rapamycin (mTOR) pathway in the l-leucine-induced up-regulation of system A amino acid transport. Biochem J.

[CR58] Pohjanen E, Thysell E, Jonsson P, Eklund C, Silfver A, Carlsson IB, Lundgren K, Moritz T, Svensson MB, Antti H (2007). A multivariate screening strategy for investigating metabolic effects of strenuous physical exercise in human serum. J Proteome Res.

[CR59] Rosendal L, Blangsted AK, Kristiansen J, Sogaard K, Langberg H, Sjogaard G, Kjaer M (2004). Interstitial muscle lactate, pyruvate and potassium dynamics in the trapezius muscle during repetitive low-force arm movements, measured with microdialysis. Acta Physiol Scand.

[CR60] Rosendal L, Larsson B, Kristiansen J, Peolsson M, Sogaard K, Kjaer M, Sorensen J, Gerdle B (2004). Increase in muscle nociceptive substances and anaerobic metabolism in patients with trapezius myalgia: microdialysis in rest and during exercise. Pain.

[CR61] Rosendal L, Kristiansen J, Gerdle B, Sogaard K, Peolsson M, Kjaer M, Sorensen J, Larsson B (2005a) Increased levels of interstitial potassium but normal levels of muscle IL-6 and LDH in patients with trapezius myalgia. Pain 10.1016/j.pain.2005.09.026 16297553

[CR62] Rosendal L, Sogaard K, Kjaer M, Sjogaard G, Langberg H, Kristiansen J (2005). Increase in interstitial interleukin-6 of human skeletal muscle with repetitive low-force exercise. J Appl Physiol.

[CR63] Rudman D, Goldsmith M, Kutner M, Blackston D (1980). Effect of growth hormone and oxandrolone singly and together on growth rate in girls with X chromosome abnormalities. J Pediatr.

[CR64] Seiler N, Dezeure F (1990). Polyamine transport in mammalian cells. Int J Biochem.

[CR65] Sjogaard G, Rosendal L, Kristiansen J, Blangsted AK, Skotte J, Larsson B, Gerdle B, Saltin B, Sogaard K (2010). Muscle oxygenation and glycolysis in females with trapezius myalgia during stress and repetitive work using microdialysis and NIRS. Eur J Appl Physiol.

[CR66] Sjøgaard G, Zebis MK, Kiilerich K, Saltin B, Pilegaard H (2013). Exercise Training and Work Task Induced Metabolic and Stress-Related mRNA and Protein Responses in Myalgic Muscles. BioMed Res Int.

[CR67] Sjörs A, Larsson B, Dahlman J, Falkmer T, Gerdle B (2009). Physiological responses to low-force work and psychosocial stress in women with chronic trapezius myalgia. BMC Musculoskelet Disord.

[CR68] Sjostrand M, Gudbjornsdottir S, Holmang A, Strindberg L, Ekberg K, Lonnroth P (2002). Measurements of interstitial muscle glycerol in normal and insulin-resistant subjects. J Clin Endocrinol Metab.

[CR69] Stallknecht B, Kiens B, Helge JW, Richter EA, Galbo H (2004). Interstitial glycerol concentrations in human skeletal muscle and adipose tissue during graded exercise. Acta Physiol Scand.

[CR70] Svensson P, Cairns BE, Wang K, Hu JW, Graven-Nielsen T, Arendt-Nielsen L, Sessle BJ (2003). Glutamate-evoked pain and mechanical allodynia in the human masseter muscle. Pain.

[CR71] Thysell E, Chorell E, Svensson M, Jonsson P, Antti H (2012). Validated and predictive processing of gas chromatography-mass spectrometry based metabolomics data for large scale screening studies. Diagnostics and Metabolite Pattern Verification. Metabolites.

[CR72] Townsend RR, Klein S, Wolfe RR (1994). Changes in lipolytic sensitivity following repeated epinephrine infusion in humans. Am J Physiol.

[CR73] Jonsson P, Johansson AI, Gullberg J, Trygg J, A J, Grung B, Marklund S, Sjostrom M, Antti H, Moritz T (2005) High-throughput data analysis for detecting and identifying differences between samples in GC/MS-based metabolomic analyses. Anal Chem 77 (17):5635–5642 10.1021/ac050601e 16131076

[CR74] Trygg J, Wold S (2002). Orthogonal projections to latent structures (O-PLS). J Chemom.

[CR75] Trygg J, Gullberg J, Johansson A, Jonsson P, Antti H, Marklund SL, Moritz T (2005). Extraction and GC/MS analysis of the human blood plasma metabolome. Anal Chem.

[CR76] Ungerstedt U (1991). Microdialysis-principles and applications for studies in animals and man. J Intern Med.

[CR77] van Hall G, Sacchetti M, Radegran G, Saltin B (2002). Human skeletal muscle fatty acid and glycerol metabolism during rest, exercise and recovery. J Physiol.

[CR78] Visser B, van Dieen JH (2006). Pathophysiology of upper extremity muscle disorders. J Electromyogr Kinesiol.

[CR79] Wibom W, Surowiec I, Mörén L, Bergström P, Johansson M, Antti H, Bergenheim A (2010). Metabolomic patterns in glioblastoma and changes during radiotherapy—a clinical microdialysis study. J Proteome Res.

[CR80] Yan B, A J, Wang G, Lu H, Huang X, Liu Y, Zha W, Hao H, Zhang Y, Liu L, Gu S, Huang Q, Zheng Y, Sun J (2009). Metabolomic investigation into variation of endogenous metabolites in professional athletes subject to strength-endurance training. J Appl Physiol.

